# A Retrospective Analysis of Universal Health Insurance Policy‐Making Process in Iran: A Qualitative Study

**DOI:** 10.1002/hsr2.70192

**Published:** 2024-11-11

**Authors:** Mohammad Javad Kabir, Alireza Heidari, Mansoureh Lotfi, Zahra Khatirnamani, Reza Golpira

**Affiliations:** ^1^ Health Management and Social Development Research Center Golestan University of Medical Sciences Gorgan Iran; ^2^ Rajaie Cardiovascular Medical and Research Center Iran University of Medical Sciences Tehran Iran

**Keywords:** Iran, policy making, universal health coverage, Universal Health Insurance

## Abstract

**Background and Aims:**

Universal Health Insurance (UHI) coverage began in 2014, after the implementation Health Transformation Plan (HTP) to reduce out‐of‐pocket payments and improve access to health services in Iran Health Insurance Organization (IHIO). The purpose of this study was to analyze the UHI policy‐making process in Iran.

**Methods:**

This study was performed using Walt and Gilson's (1994) policy triangle framework and Kingdon's multiple streams model. Data were collected through document review, in‐depth individual interviews (constituting 17 policymakers and top, middle, and operational level managers), and one round‐table discussion (constituting 10 top managers and senior policymakers). The majority of the participants were MD and MPH (47.07%). Document review data were analyzed using thematic content analysis. Interviews and discussions were analyzed using framework analysis.

**Results:**

The high‐level documents of Iran emphasize the need for insurance coverage. The commitment of the new government to enforce the laws, concurrent implementation of the UHI and the HTP, the new government's approach to enforcing civil rights, and media pressure to speed up the implementation process. UHI was initiated in 2014 and about 11 million people across the country were covered by free health insurance but the budget did not change in proportion to the increase in insured population, and increased IHIO's debt to contracting centers.

**Conclusions:**

To achieve continuity and effectiveness of UHI in Iran, all stakeholders should be involved. Political will and commitment and sustainable financing are needed for it.

## 1 Introduction

One of the goals of health systems is to protect people from the financial costs of disease by reducing or eliminating out‐of‐pocket (OOP) payments [1]. The success rate of health systems is of great importance for policymakers. Achieving fairness in provision of care is challenging since illness, disability, premature death, and mortality are more prevalent in people with lower socioeconomic status [2]. In this regard, at the 58th World Health Assembly, member states were urged to ensure that health financing systems introduce prepayment mechanisms for health care to share risk among the population and to avoid catastrophic health expenditure (CHE) and impoverishment of individuals as a result of receiving care. Following this call, the 2010 Universal Health Coverage (UHC) is one of the targets of Sustainable Development Goals which means that all individuals can receive the appropriate preventive, curative, and rehabilitative care without suffering financial hardship [3, 4, 5].

Low‐ and middle‐income countries (LMICs) have inefficient prepaid, tax, and social health insurance systems [6]. These countries are currently considering how to improve their healthcare systems so that they can provide effective financial protection for everyone as part of the UHC [5]. UHC has two main goals: financial risk protection and access to needed care [7]. It is an important way to expand access to effective healthcare services, reduce financial hardship during illness, and improve health outcomes. Many other middle‐income countries have tried to address inequalities in access to health care and health outcomes through UHC by introducing health insurance schemes and health system‐strengthening programs [8].

The Islamic Republic of Iran is located in the Eastern Mediterranean region with a population of more than 80 million [9]. Organizational Structure of the Iranian healthcare system includes primary, secondary, and tertiary levels. Health insurance, Government funds, general taxation, individual donations, and OOP are sources of health financing. Health insurance coverage is provided by SHI, institutional health insurance funds, and commercial organizations in Iran [10].

Before the Universal Health Insurance (UHI), the studies show that the share of OOP payments from total health expenditures in Iranian households more than 50% [11, 12]. High OOP share of total health expenditures increases the risk of households incurring CHE [13]. Studies have shown that CHE rate in Iran is between 6% and 24%. This evidence confirms the unacceptable level of financial support for Iranian patients [9]. In 2000, the World Health Organization (WHO) ranked Iran 93rd out of 191 countries in terms of overall level of health and 112th in fairness of financial contribution (WHO 2000). Poor performance in fairness of financial contribution reflects the fact that many households lack health insurance or financial support for the cost of illness, and therefore OOP payments are common [14, 15].

Equitable access to health care for all Iranian citizens has been emphasized as a right in Article 29 of the Iranian Constitution [9]. In this regard, there is a long history of efforts to remove financial barriers to health care for all. Actions taken include the passage of the Social Security Act of 1975, the creation of the Health Network in 1984 to ensure equal and equitable access to primary health care, the passage of the Health Insurance Act of 1994 to cover the entire population until 1999, and the adoption of the Family Physician Act of 2004 [13]. Despite the implementation of these important policies over the last few decades, progress toward achieving UHC has not been very satisfactory. Despite repeated emphasis in high‐level documents (e.g., 3rd to 6th Five‐Year Development Plans) on the need to expand health insurance coverage to all Iranians, reduce OOP payments, and promote equitable access to health care, many people still remained without coverage and had to pay OOP for all medical expenses. To address the challenges in health financing, a series of policy interventions called the Health Transformation Plan (HTP) were implemented in May 2014, mainly to increase the share of the health sector from global funding. The purpose of this plan was to reduce OOP costs and financial burden in public health by lowering coinsurance rates in the public sector and provision of free health insurance coverage. To achieve this goal, a new subfund was created in the Iran Health Insurance Organization (IHIO) for those who are covered free of charge by the government [10]. Implementation of free insurance coverage for uninsured people through the HTP has increased coverage to more than 95% [16].

Although some studies have addressed the challenges of implementing UHC [10, 17], but specifically did not focus on UHI after HTP. Therefore, this study was conducted to analyze to better understand the process of policymaking UHI policy‐making process in Iran.

## 2 Materials and Methods

This Quantitative study is based on deductive reasoning. The study was performed using Walt and Gilson's triangle and Kingdon's multiple streams models. Walt and Gilson's triangle framework consists of four elements: content, context, actors, and process. Content includes policy objectives, operational policies, legislation, regulations, and so forth. Actors refer to influential individuals, groups, and organizations. Context refers to social, economic, political, cultural, and other environmental conditions. Process consists of four parts: agenda setting, policy formulation, policy implementation, and policy evaluation [18]. Kingdon's multiple streams model shows the policies agenda‐setting. Based on this model, the interaction of three streams problems, policies and politics can lead to a policy window [19].

The participants in this study included 17 policymakers and top, middle, and operational level managers of the IHIO and the MoH and Ministry of Welfare of Iran who were selected by purposive and snowball sampling techniques. Criteria for sample selection included having the necessary knowledge and information, active participation in the development and implementation of UHI in Iran, and interest in participating in the research.

Data were collected via document review, semistructured interviews, and round‐table discussion.

To collect the documents, scientific databases, and internal websites were reviewed purposefully. Documents for the study were original documents, survey reports, and national meeting reports of organizations involved in UHI.

Semistructured interviews were conducted using an interview guide. The questions in the interview guide include issues of policy context, content, policy‐making process (policy agenda, policy formulation, policy implementation, policy evaluation), and stakeholders' engagement of UHI. The interviews were conducted by one of the authors with experience in qualitative studies and PhD degree and he had no employment relationship with the IHIO.

The interviews were conducted face‐to‐face at the appointed time and place. The interviews lasted between 35 and 65 min. At the beginning of each interview, the interviewees were given a summary of the research topic and an explanation on how to the data would be used. The interviews were recorded after obtaining permission from the interviewees and assuring them that their personal information would remain confidential. Notes were taken during the interviews, and each interview was recorded and transcribed. The obtained data were provided to the participants to verify the accuracy of the results. The interviews continued until the saturation level was reached and no new data were presented.

To enhance the validity of the study, 1 round‐table discussion were held in which 10 top managers and senior policymakers participated.

Lincoln and Guba's criteria of credibility, dependability, confirmability, and transferability criteria were used to ensure trustworthiness [20, 21, 22]. For credibility, the participants reviewed and confirmed the findings of the study. For transferability, participants' descriptions and direct quotations were used. For dependability, the initial codes and examples of how to extract the primary and secondary codes were provided to an external observer. To confirm the correctness of the research method, the transcript of a number of interviews, the extracted codes, and categories were provided to the research colleagues and a number of faculty members who were familiar with qualitative analysis and were not part of the study.

### 2.1 Data Analysis

Data were analyzed using framework analysis, which consists of five steps: (1) familiarization; (2) identifying a thematic framework; (3) indexing; (4) charting; and (5) mapping and interpretation. Thematic content analysis was used to analyze documents for validating the findings of the interviews and discussions.

### 2.2 Ethical Consideration

The ethics committee of Golestan University of Medical Sciences granted the ethical approval (ethics code: IR.GOUMS.REC.1400.434). Ethical considerations of the study include the anonymity of individuals and their administrative role, respecting the rights of each participant, explaining the purpose and nature of the research to the participants, and using the transcripts verbatim without any changes. Verbal consent was obtained from all participants. All methods were carried out in accordance with relevant guidelines.

## 3 Results

A total of 17 participants were interviewed, of whom 15 (88%) were male and 2 (12%) were female. Ages ranged from 51 to 67 years, with an average of 56.67 years. Experience ranged from 15 to 35 years, with an average of 23.76 years. Nearly half of the participants were MD and MPH (47.07%). The participants' demographic characteristics are presented in Table 1.

**Table 1:** The participants' demographic characteristics.

Characteristic	Percentage (%) of total (*N* = 17)
Gender	
Male	88 (15)
Female	12 (2)
Age	
Mean (SD)	56.76 (3.61)
Range	51–67
Years of experience	
Mean (SD)	23.76 (4.55)
Range	15–35
Expertise	
Government management	5.88 (1)
Information technology engineering	17.65 (3)
MD and MPH	47.07 (8)
Medical specialist	17.64 (3)
Health policy	5.88 (1)
Health care service management	5.88 (1)

**Figure 1:**
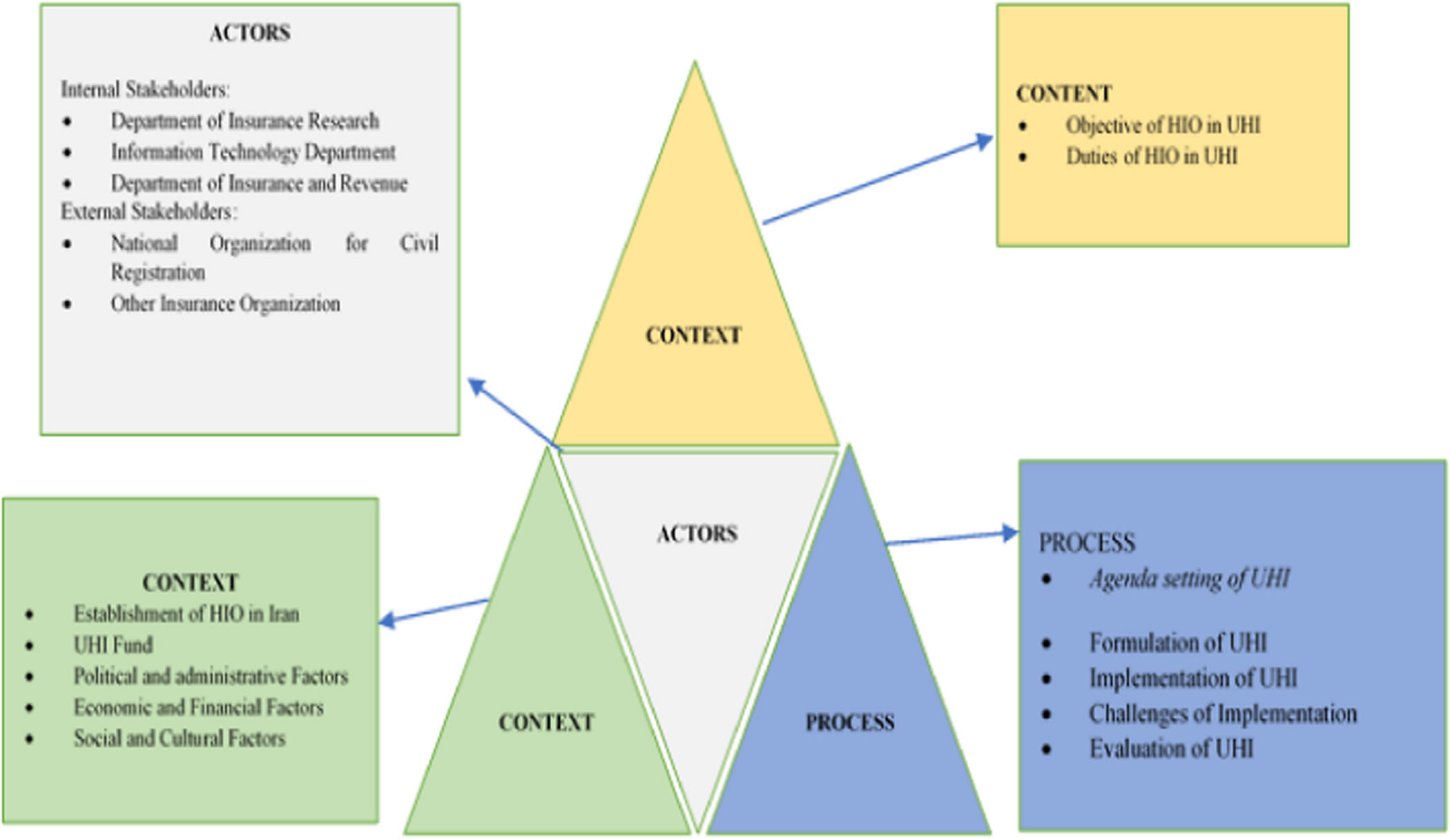
The policy framework of UHI in Iran based on Walt and Gilson framework.

Findings were reported based on four categories of policy triangle framework (context, content, process, and actors). These categories are explained as follows (Figure 1):

### 3.1 Context

IHIO was established as a result of the UHI Act, which was approved by the Parliament in 1995. Article 38 of the Law on the Fifth Development Plan emphasizes the need to make universalize basic insurance, and it was the responsibility of the IHIO to ensure universal coverage.

UHI Fund is one of the funds of the IHIO of Iran. Other funds in this organization include the Government Personnel Fund, Special Groups Fund, Rural Households Fund, UHI Fund, and Health Insurance Fund.

#### 3.1.1 Political and Administrative Factors

The high‐level documents of the Islamic Republic of Iran (the Constitution, Fifth and Sixth Development Plans, and policies announced by the Supreme Leader) emphasize the need for insurance coverage, but management instability and dependence of programs and policies on individuals have hindered the implementation of the UHI. Factors such as insufficient manpower, increased staff workload, lack of accurate estimates of the uninsured population, and lack of a single database for all insurance organizations have created challenges for implementation process.

Lack of a single database of the insured population by all insurance organizations and the resulting overlap is one of the main problems that still exist. This is due to the lack of cooperation between insurance organizations and the IHIO (A senior manager of the IHIO)

Unfortunately, many changes in the country depend on individuals and are not systematic reforms. A person at the head of the MoH has been able to persuade the government and the parliament to run the program and the Plan and Budget Organization to pay it (A senior manager of a health insurance organization)

#### 3.1.2 Economic and Financial Factors

Insufficient financial resources, not aggregating resources in the IHIO, unreasonable premiums, inefficient payment system, and lack of allocation and full distribution of credits poor allocation of funds are some of the economic and financial factors affecting the execution of UHI.

According to the UHI Act, our service package is designed based on the real price and the real insurance premium per capita. Neither our service package has a real price, nor is our premium per capita determined based on the real price or income. The UHI Act states that the basic insurance package should be based on the actual price, not per capita. So, there is always a gap between service and per capita. There is always a deficit in the system (A senior director of the IHIO)

We were supposed to be paid per capita for the population to be insured, but this per capita was not paid to us. We insured a large number of people without any per capita payment to the organization (A senior director of the IHIO)

#### 3.1.3 Social and Cultural Factors

Most of the participants mentioned factors such as the need for culture building, acceptance of the program by the people, and people's involvement as key social and cultural factors influencing the implementation of UHI.

We can make people aware of their rights and they can help us with that. Before building any infrastructure, we need to build a conducive culture. If we bring in a lot of money, but people are not ready to accept the program, it will fail (A senior director of the MoH)

### 3.2 Content

According to the 3rd, 29th, and 43rd Principles of the Iranian Constitution, the government is obliged to ensure the universal right to health for all members of society. According to the UHI Act of 1994, the entire population, especially the disadvantaged, should be covered by health insurance by the government within 5 years. Moreover, Section A of Article 38 of the Law on the Fifth Development Plan, Section A of Article 70 of the Law on the Sixth Development Plan, and general health policies announced by the Iranian Supreme Leader in 1993 emphasize the universalization of basic health insurance.

Most of the participants considered the IHIO was assigned with the task of undertaking population coverage and financial protection, and UHI was initiated in 2014 at the same time as the HTP to reduce OOP payments and increase access to health services. In the first phase, about 11 million people across the country were covered by free health insurance, but the budget did not change in proportion to the increase in insured population, and increased IHIO's debt to contracting centers. The Council of Ministers approved the executive regulations of Section A of Article 70 of the Law on the Sixth Development Plan titled “Regulations on Compulsory Basic Health Insurance and Household Means‐Testing.” Accordingly, the government was obligated to give health insurance to the entire population based on household means‐testing.

### 3.3 Process

#### 3.3.1 Agenda Setting

##### 3.3.1.1 Problem Stream

Most of the participants considered the large uninsured population, high OOP payments and CHE rate, failed attempts at full population coverage in previous years, lack of managerial commitment to implementation of laws, and lack of proper enforcement and transparency as problems.

We've been insuring people ever since the establishment of the IHIO in 1995, but we didn't insure more than 8 million people in total, which means that we've failed at universal health coverage for 20 years (A senior manager of the IHIO)

Many people weren't affiliated with any government organization. They're mostly business owners or self‐employed. These groups paid a higher premium than others and faced some restrictions in receiving services. They had to wait 3 months from the date of their insurance coverage to be allowed to receive inpatient services. In addition, at that time they paid a higher fee for inpatient services and wouldn't be reimbursed if they didn't go to government centers (An executive director of the IHIO)

OOPs were high before the implementation of UHI. Catastrophic health expenditures were significant. Our problem isn't the lack of laws and regulations. The problem is lack of commitment to law enforcement (A senior health insurance executive)

From the beginning of the Fifth Development Plan, we've been obligated to provide universal insurance coverage, but policymakers haven't established proper enforcement mechanisms for implementing it (A senior director of the MoH)

##### 3.3.1.2 Policy Stream

To address the problem of the uninsured population, urban inpatient care card, rural population insurance, and Iranian insurance were implemented between 2001 and 2013, but these schemes did not meet expectations in terms of quantity and service coverage.

Following Rouhani's presidency in 2014 and simultaneously with the HTP, the implementation of UHI began in Iran. Feasibility, quick win, and people's satisfaction with the UHI compared with other government programs were some of the reasons why the government was willing to implement it. In addition, the new government decided to eliminate part of the people's cash subsidies and allocate it to public health.

The president faced many problems in various areas. The government alone couldn't reform the economy and the banking system and solve the problems caused by sanctions, and these processes took a long time. It was felt that intervention in health insurance is easier than other fields. It leads to quicker result. A lot of people benefit from it. Public satisfaction increases (A MoH policymaker)

The government had just taken over and they wanted to encourage people not to receive cash subsidies. They said that if people do not receive these subsidies, they would spend the money on health, and introduced UHI as a supplementary package for people who didn't receive subsidies (A senior director of the IHIO)

##### 3.3.1.3 Politic Stream

The commitment of the new government to enforce the laws, concurrent implementation of the UHI and the HTP, the new government's approach to enforcing civil rights, and media pressure to speed up the implementation process led to UHC being placed on the agenda.

We had not implemented UHC laws before Rouhani president and the 11th government came to power. In 2013, the Minister of Health proposed the HTP in the process of approving the 2014 budget. We also had Section A of Article 38 of the Fifth Development Plan, which states that there should be no uninsured person in Iran (A senior director of the MoH)

After the interview of the CEO of the IHIO at the time, the media got involved. Public awareness increases and the UHI became a public demand. There was pressure to complete the program by a certain date (A senior director of the IHIO)

The new government's approach was to enforce citizenship rights. Paying attention to the demands of the society was one of the priorities of the government because many of these demands had not been addressed by the previous government (A MoH policymaker)

#### 3.3.2 Policy Formulation

The UHI was approved on April 8, 2014 at the Targeted Subsidies Center and was communicated to the IHIO for implementation the following day. The IHIO was not ready to immediately implement the program due to its outdated hardware and software infrastructure.

Without taking into account the technical requirements and infrastructure needed to implement the program, the CEO of the organization at the time said on Thursday that people could come to register on Saturday, but we weren't fully prepared. We thought that the process of approving the plan would take a long time and we would have the opportunity to make executive arrangements. Besides, registration was free; if it weren't free, our job would be much easier because there would be very few applicants (An executive director of the IHIO)

To check the requirements for the implementation of the UHI, a meeting was held in the IHIO on the weekend. The CEO, the deputy director, senior managers, and related experts participated in this meeting and it was decided to ask the Minister of Welfare for a 10‐day deadline to develop the necessary software infrastructure. Subsequently, several meetings were held at the expert level.

#### 3.3.3 Policy Implementation

After the 10‐day deadline, the UHI was made public. A top‐down approach was taken to implement the plan. One of the senior managers of the IHIO mentioned the lack of time to launch the program as one of the reasons for adoption of such an approach.

We were so short of time that we couldn't coordinate with our local offices before starting the program (A senior manager of the IHIO)

To register the applicants, a system was operationalized within about 7 days. Applicants registered their information in the system and the identity of the applicants was verified with the help of the National Organization for Civil Registration. The applicants' information was also matched against the information from other insurance organizations, and if the applicant did not have basic insurance, an insurance card would be issued for them. Public service offices were selected to issue insurance cards. Since there were a large number of applicants for free insurance in the early days of the program, a 24‐h headquarters was set up at the IHIO for a week.

Six months after the implementation of the HTP, the guideline titled “Relative Value of Health Services” was edited to be include realistic prices and eliminate informal payments. This review resulted in a 225% increase in medical fees, but the allocated financial resources were not commensurate with the amount required, and the IHIO faced a large deficit. The reasons for this deficit were the allocation of resources to insured inpatients and not to outpatients, the increase in the number of full‐time faculty members in public hospitals, and the increase in the provision of services with higher reimbursement rate. The sharp drop in oil prices also led to a decline in government revenues and its inability to allocate the required financial resources for subsequent years. Adding a large number of pharmaceutical items to the basic insurance package without a guideline added to this challenge.

The increase in fees caused the system to collapse. The IHIO was faced with obligations that were in excess of its resources, and unfortunately the warnings of the organization's experts were not heeded (An executive director of the IHIO)

In some cases, the required financial resources were provided to the MoH, and it was decided that the MoH finance UHI from the HTP funds, but it never fully materialized (A senior manager of the IHIO)

About 300 pharmaceutical items were added to the basic insurance package without any guidelines. We didn't have accurate estimates of its induced demand and the increase in the number of referrals (A senior manager of the IHIO)

#### 3.3.4 Policy Evaluation

The results of the program were evaluated by monitoring and analyzing the database, receiving reports from IHIO's local offices, and conducting studies to evaluate the results of the program. Coverage of about 10 million uninsured people, reduction of OOP payments, and IHIO's software system reform were the positive results of implementing the national UHI policy.

We conducted several studies and analyzed the data from our database. We finally insured 10 million disadvantaged people. These people received free services. OOPs decreased and IHIO covered these costs. Conditions were created that allowed us to change the entire IT system of the organization and centralize our decentralized databases (Executive Director of the IHIO).

Despite these achievements, the implementation of this policy also faced challenges. The process of issuing insurance cards was hindered by the large number of applicants referring to public service offices in the most populous provinces for registration. No call center was established to answer the questions and solve the problems of insurance applicants. Some managers and employees resisted the new system. Following free coverage of the applicants, some insurance organizations transferred their high‐cost applicants to the IHIO. In addition to these problems, universal insurance was not mandatory.

Insurance organizations transferred their high‐cost applicants to the IHIO. These people did not pay insurance premiums either, even though we asked the Ministry of Welfare to identify people's ability to pay so that we can charge them accordingly, but this never happened (Senior Director of the IHIO)

We insured 14 million people back then, but now only 9 million people are covered. A lot of people say to themselves, ‘why do I need to be covered now, I can insure myself whenever I want; it's free, too’. Unfortunately, we didn't have the authority for mandatory coverage (Senior Policymaker of the IHIO)

### 3.4 Stakeholders

IHIO has been in charge of implementing the UHI in Iran. Several departments within the organization were complicated in the execution of this plan, including the Department of Insurance Research, Information Technology Department, and the Department of Insurance and Revenue, as well as all the corresponding departments in its local offices. A private company was contracted to design and support the applicant registration system, and public service offices across the country were responsible for issuing insurance cards.

Among external stakeholders, the National Organization for Civil Registration verified the identity of the insured people relatively quickly and accurately. However, other insurance organizations were not cooperative and did not immediately provide the IHIO with information about their covered population to eliminate overlap and help create a single database.

We felt that the insurance companies were a bit reluctant to cooperate with us. We barely got any information from the Social Security Organization. They didn't provide information in a timely manner. Other insurance organizations gave us information after a great deal of correspondence, several meetings, many follow‐ups, and with the help of intermediaries. Banks didn't provide information. Some insurance organizations didn't provide any information due to legal restrictions. The Armed Forces Insurance Organization stated that the information of its insured was confidential (Senior Director of the IHIO)

## 4 Discussion

According to Iran National Health Accounts (NHA), OOP payments in are higher than the global average, and to address this problem, Iran development plans emphasize the quantitative and qualitative development of health insurance [23]. In this regard, various insurance plans such as inpatient care card, rural population insurance, and Iranian insurance were designed and implemented, but these plans were not very successful to achieve the goal of expanding UHI. Ebrahimipour et al. mentioned that there is not cross subsidization in Iran and that the people covered by these funds are mostly poor people who have less financial protection against costs of illnesses [14]. While countries that have succeeded in achieving UHC have accepted the principle of fairness and universality of services, they have abandoned approaches to collecting premiums from the very poor [24].

In Japan, for example, almost everyone was insured in 1961. The copayment rate is the same for everyone excluding the elderly and children. Equity has been achieved by allocating subsidies from public revenue to plans that enroll low‐income people, and by implementing cross‐subsidization among elderly health care funding plans [25]. Also, Thailand's insurance system, with government support and a sufficient budget, covered 75% of the population in 2002 by implementing voluntary health card schemes and a social security scheme [26, 27]. However, the national health insurance in Korea was launched with a low benefit package and benefit coverage gradually increased. The government prioritized the expansion of population coverage because expanding benefit coverage could hinder population coverage [28].

The 11th government launched the UHI implementation to reduce uninsured people and OOP payments by the Health Insurance Organization of Iran freely. The commitment of executives to implement the program was one of the key factors in launching and implementing the program. Similarly, Yu cited the political commitment of senior leaders as one of the key factors in China's success in this field [29]. Government capacity and political will have been identified as key factors in the development of healthcare financing policies and UHC promotion in the Philippines, Taiwan, and Thailand [30, 31, 32]. Without government subsidies for workers in the informal sector, UHC in many LMICs seems impossible. Good governance, transparency, and accountability in the health insurance program as well as the design of the payment system and the regulations for health care providers all need serious government support [28].

The UHI was approved at the national level and it was announced publicly. Ham and Hill have proposed “bottom‐up” and “top‐down” approaches to policy implementation. The main difference between these approaches is the degree of involvement of actors in the political cycle [33]. In the “bottom‐up” approach, actors can influence performance through negotiation and interaction, while in the “top‐down” approach, bargaining and interaction between different levels of the policy cycle are low [5, 34]. The “top‐down” approach—the approach used to implement the UHI in Iran—focused on a small group of high‐level policymakers in the IHIO of Iran who issued mandates to low‐level officials in local organizations for implementation.

Insufficient financial resources were one of the obstacles to promotion of the UHI in Iran, which is consistent with the results of Ibrahimipour et al. [14] Most developing countries are also affected by this problem. These countries have large informal populations and face challenges in collecting taxes and premiums. Government expenditure on health has increased in most of these countries: between 5% and 11% in Ghana, Indonesia, Rwanda, and Vietnam; and between 1% and 3% in India, Kenya, Mali, and Nigeria. Countries that provide significant targeted subsidies (Ghana, India, Indonesia, Philippines, and Vietnam) are increasingly using tax revenue to fund coverage expansion. Despite the difficulties in collecting income, some countries (Kenya, Philippines, Nigeria, Rwanda, and Ghana) have tried collecting voluntary premiums from households [35]. These countries choose to direct funds to where they need it the most. For example, performance‐based funding schemes were made integral components of their UHC insurance policies, and these tend to increase the coverage in specific areas (usually maternal and child health) [36].

In recent years, however, some countries have been able to achieve a desirable level of UHC, which indicates the possibility of achieving UHC even in the face of financial shortages. Turkey and Thailand are two of these countries [37]. An analysis of the establishment of UHC in Thailand indicates that financial and political commitment, appropriate social services, the use of research evidence, and the presence of a capable purchasing organization prepared the ground for achieving this important goal [26, 27]. In addition, a study of UHC in Turkey shows that political commitment, a strong electronic registration system, strengthening of the central system of the MoH in terms of technical capacity and staffing, public participation in the implementation of the program, and appropriate incentives for reducing resistance to change are key factors in the success of this country toward UHC [38, 39].

Culture building, especially in more deprived areas, is one of the effective sociocultural factors for achieving universal insurance coverage. Poor people should know their rights and use free health insurance. Education can increase awareness and improve people's attitudes [40]. In this regard, national and provincial media can play an useful role.

To evaluate the UHI implementation, several studies confirmed the reduction of OOP payments and the reduction catastrophic costs [41, 42, 43, 44]. Although the population covered by the Self‐Employed and Iranians Fund has increased significantly since 2014 and simultaneously with the UHI implementation, but the population of the funds of government employees, villagers, nomads, and others has decreased. The decrease in the number of insured people, especially in the government employees' fund, is due to the reduction of official employment of government employees, the gradual departure of covered people, and the obligation to pay 15% per capita by Insured persons of Iranian Insurance Fund and unwillingness to pay insurance premiums except in serious illnesses and emergencies [45]. Although this policy was able to insure many people, but according to statistics from the 2016 general census in Iran, about 10% of people were still not covered by any health insurance [46]. In this regard, a recent report by the World Bank based on a study conducted in 24 middle‐income and high‐income countries, emphasized that insurance coverage will not reach 100% without coercion [47].

Some strengths of the study is using the qualitative approach that focuses on the policymakers and managers using the triangulation strategy. The two well‐known conceptual frameworks that are used, that is, the policy triangle framework and Kingdon's three‐stream model, can gather data on the process, content, context, and actors for policy analysis. However, this study also had its restriction. This study was a cross‐sectional study and not many public documents were available to investigate. The patients did not have enough information to answer the interview guide questions about policy analysis. Therefore, regarding the effect of the implemented policy on the people, we quoted from other participants.

## 5 Conclusion

This Research was conducted to study UHI policies in Iran. These studies helped identify the various components of the policy cycle and the factors influencing policy formulation, implementation, and evaluation. There is no single way to provide health insurance coverage for the entire population. Providing access to health services and financial protection against health costs for all people is a long process and this policy should remain a permanent priority for governments. Involving all stakeholders and addressing economic and operational challenges can result in the continuity and effectiveness of such programs. political will and commitment, sustainable financing and management of health system financial resources through the insurance system can result in continuity and effectiveness of HUI. To carry out future studies, it is suggested to determine the appropriate strategies for the quantitative and qualitative development of health insurance and to determine the effect of insurance policies from the people's point of view.

## Author Contributions

**Mohammad Javad Kabir:** Conceptualization, data curation, formal analysis, methodology, project administration, writing–original draft, funding acquisition, visualization, supervision. **Alireza Heidari:** Conceptualization, data curation, investigation, methodology, writing–original draft, writing–review and editing, validation, software. **Mansoureh Lotfi:** Writing–original draft, conceptualization, data curation, methodology. **Zahra Khatirnamani:** Writing–original draft, conceptualization, data curation, investigation, methodology. **Reza Golpira:** Writing–original draft, methodology, investigation, resources. All authors have read and approved the final version of the manuscript.

## Conflict of Interest

The authors declare no conflict of interest.

## Transparency Statement

The lead author Alireza Heidari affirms that this manuscript is an honest, accurate, and transparent account of the study being reported; that no important aspects of the study have been omitted; and that any discrepancies from the study as planned (and, if relevant, registered) have been explained.

## Data Availability

Data will be made available upon reasonable request. Corresponding author had full access to all of the data in this study and takes complete responsibility for the integrity of the data and the accuracy of the data analysis.
